# Effects of Nonmagnetic Zn^2+^ Ion and RE Ion Substitution on the Magnetic Properties of Functional Nanomaterials Co_1−y_Zn_y_RE_x_Fe_2−x_O_4_ (RE = La, Sm, Gd) by Sol–Gel

**DOI:** 10.3390/molecules28176280

**Published:** 2023-08-28

**Authors:** Jinpei Lin, Xingxing Yang, Kaimin Su, Fang Yang, Yun He, Qing Lin

**Affiliations:** 1 College of Biomedical Information and Engineering, Hainan Medical University, Haikou 571199, China; 2 College of Physics and Technology, Guangxi Normal University, Guilin 541004, China; 3 Department of Civil Engineering, Jiangxi Water Resources Institute, Nanchang 330013, China

**Keywords:** nonmagnetic, functional materials, structure, Mössbauer, substitution, sol–gel

## Abstract

Magnetic Functional Nanomaterials Co_1−y_Zn_y_RE_x_Fe_2−x_O_4_ (RE (rare-earth) = La,Sm,Gd) were prepared using the sol–gel combustion method. XRD characterization confirms that the ferrite samples we synthesized are single-phase cubic structures. The variation in the average crystalline size and lattice parameter is related to RE ion doping. The Mössbauer spectra of CoRE_x_Fe_2−x_O_4_ are two sets of magnetic six-wire peaks that indicate the ferrimagnetic behavior of the sample. The calcination temperature greatly influences the absorption area of Mössbauer for CoFe_2_O_4_, indicating that the calcination temperature affects the iron ion content at the octahedral B and tetrahedral A sites. Additionally, scanning electron microscopy measurements of the substituted specimens reveal that the ferrite powders are nanoparticles. With an increase in RE ions, the coercivity increases, and the saturation magnetization changes obviously. The XRD characterization of Co_0.7_Zn_0.3_La_x_Fe_2−x_O_4_ shows that the main crystalline phase of the sample is the cubic spinel structure phase, and there are fewer secondary crystalline phases. The lattice parameter tends to decrease with the substitution of La^3+^ ions. The average grain size decreased significantly with the increase in La content. From ferrimagnetic state transition to relaxation behavior, the hyperfine magnetic field decreases in La concentration by room temperature Mössbauer spectra. With the substitution of La^3+^ ions, both the saturation magnetization and coercivity of the samples were reduced, and the coercivity of all samples was lower.

## 1. Introduction 

CoFe_2_O_4_ is an important magnetostriction and magnetic material [1,2,3,4]. CoFe_2_O_4_ has been widely used as magnetic recording material [5,6,7,8]. Mössbauer effect spectra are a suitable technique to detect the local environment of iron nuclei around various divalent cations consisting of a close-packed oxygen arrangement [9,10,11,12,13]. The rare-earth ions exhibit interesting magnetic and magnetostrictive properties [14,15,16,17,18], playing an essential role in the magnetocrystalline anisotropy of ferrite. The RE ions of 4f elements substitute Fe^3+^ ions of 3d elements in ferrites, exhibiting the strong 3d–4f spin–orbital coupling [19,20,21,22,23]. Kumar et al. [10] studied the magnet anisotropy on the La^3+^ substitution effect of CoFe_2−x_La_x_O_4_ ferrites. Mariano et al. [11] synthesized the magnetic nanoparticles Fe_2−x_CoSm_x_O_4_ and analyzed the variation of physical and magnetic properties with Sm content. Simultaneously, Rana et al. [12] investigated the dielectric properties, revealing a strong dependence on Gd^3+^ substitution in nano cobalt ferrite. Mixed ferrite has vast applications in a wide range, from frequencies to radio microwaves [24,25,26,27]. They play an irreplaceable role in magnetic recording, computer memory, and microwave devices due to their low eddy current loss and high resistivity [28,29,30,31]. In nanomaterials containing transition metals with 3D band structures, magnetic carriers are 3D shell electrons that migrate from one atom to another [32,33,34,35]. The magnetic moments are localized in individual atoms [36,37]. The dielectric constants and permeability of Co_0.5_Zn_0.5_Fe_2_O_4_ in the range of 10 MHz to 1.0 GHz show the potential of the material as an electromagnetic interference absorber [38]. For Co_1−x_Zn_x_Fe_2_O_4+γ_ of the composition x = 0.6, its paramagnetic transition temperature is 310–334 K, indicating that magnetic fluid hyperthermia is suitable for self-control states [39]. For synthesizing nanometer ferrite powder, the sol–gel method is a simple and economical process.

To sum up, the substitution of nonmagnetic Zn^2+^ ions and rare-earth ions will have a greater impact on magnetic properties. The above literature mainly studies the influence of Zn ion or rare-earth ion single-component doping on sample properties, while some of the literature has focused on the influence of co-doped cobalt ferrite with nonmagnetic Zn ions and rare-earth ions on sample magnetism. Therefore, the co-doping of nonmagnetic Zn ions and rare-earth ions will be studied in this paper. It has many advantages, for example, inexpensive raw material, low external energy consumption, homogenous highly reactive powder, and good crystallinity [40]. In this paper, Co_1−y_Zn_y_RE_x_Fe_2−x_O_4_ (RE = La, Sm, Gd) functional nanomaterials were prepared by sol–gel auto combustion method, and the effects of nonmagnetic zinc ion and rare-earth ion substitution on their structural magnetic properties were studied.

## 2. Results and Discussion

### 2.1. The XRD Analysis

**Figure 1:**
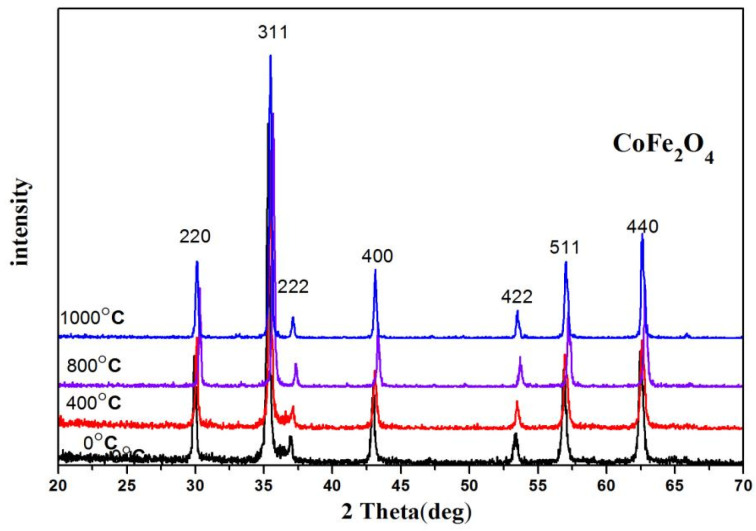
XRD diffraction patterns of the CoFe_2_O_4_ unsintered and sintered at 400, 800, and 1000 °C.

Figure 1 shows the XRD diffraction pattern of CoFe_2_O_4_ sintered at different temperatures, from which no impurity peak is observed, confirming that CoFe_2_O_4_ possesses a single spinel-structure ferrite.The average crystallite size increased, and the lattice parameter showed changes for CoFe_2_O_4_ sintered at different temperatures, as indicated in Table 1. Previous studies [4,20,21] have shown that the diffraction peaks of XRD are not sharp for CoFe_2_O_4_ calcined at low temperatures. 

Ferrite doped with nonmagnetic rare-earth ions exhibits strong spin–orbit coupling (3d–4f) since the rare-earth ions plan an important role in determining the magnetocrystalline anisotropy [8,9,10,11]. In our results, however, the diffraction peaks of XRD are sharp for CoFe_2_O_4_ without burning. The samples without calcination still display excellent crystallinity.

**Figure 2:**
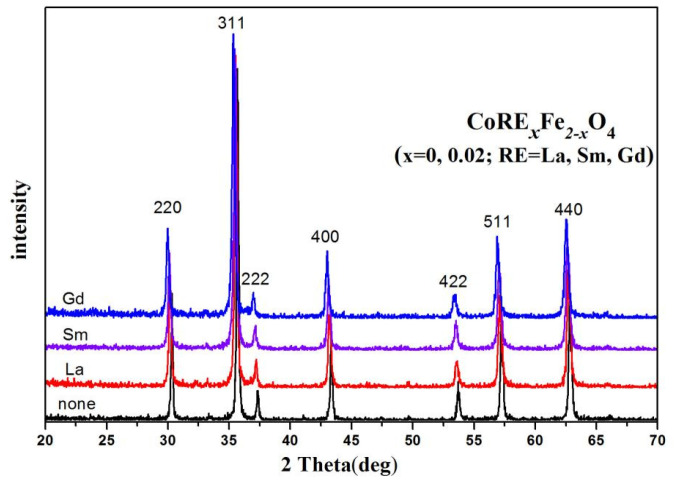
XRD diffraction patterns of CoRE_x_Fe_2−x_O_4_ (x = 0, 0.02; RE = La, Sm, Gd) sintered at 800 °C.

Figure 2 depicts the X-ray diffraction (XRD) analysis of CoRE_x_Fe_2−x_O_4_ ferrites (x = 0, 0.02; RE = La, Sm, Gd). CoRE_x_Fe_2−x_O_4_ ferrites possess a single spinel structure (JCPDS card numbers 22-1086). No other impurity peaks were observed in the XRD pattern of the sample. The lattice constant of CoRE_x_Fe_2−x_O_4_ ferrites is larger than that of CoFe_2_O_4_ ferrites due to the larger ionic radius of RE^3+^ ions (r_La3+_ = 1.03 Å, r_Sm3+_ = 0.96 Å, r_Gd3+_ = 0.938 Å) than that of Fe^3+^ ions (0.645 Å) [10,12,13,14]. Table 2 presents the corresponding data. The average crystallite size of the investigated samples, CoRE_x_Fe_2−x_O_4_ (x = 0, 0.02; RE = La, Sm, Gd), estimated by the Debye–Scherrer formula [7,13], ranges from 37.4 nm to 55.6 nm. When RE ions are employed as substitutes, the average crystallite size decreases, which aligns with other reports of the literature [14,15,16].

The XRD density is calculated using the formula [17,18,19]:

(1) $$\rho_{x}=\frac{8M}{Na^{3}}$$

where *M* is the relative molecular mass, *N* is Avogadro’s number, and a is the lattice parameter. The XRD density decreases with RE^3+^ substitution, as shown in Table 2. Doping RE ions leads to an increase in relative molecular mass, and according to Equation (1), the lattice parameter will also increase. The decrease in XRD density can be attributed to the increasing lattice parameter.

**Figure 3:**
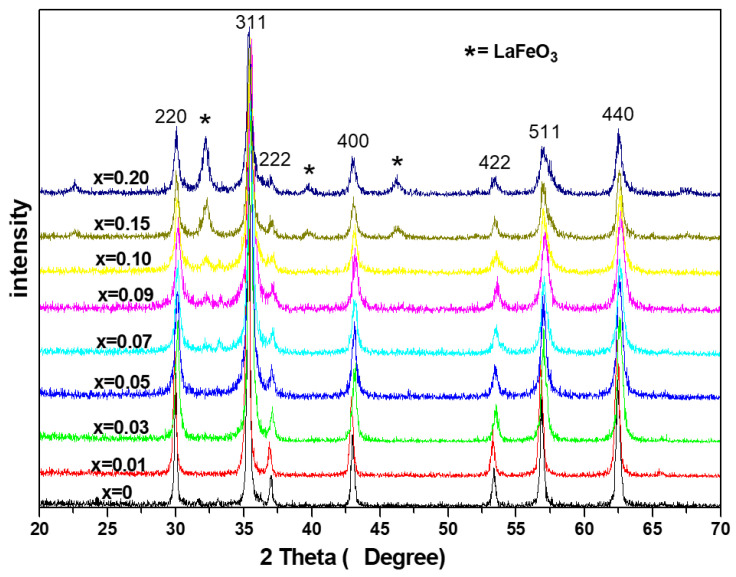
XRD diffraction patterns of Co_0.7_Zn_0.3_La_x_Fe_2−x_O_4_ calcined at 800 °C.

Figure 3 displays the XRD patterns of Co_0.7_Zn_0.3_La_x_Fe_2−x_O_4_ (x = 0~0.20) ferrites calcined at 800 °C for 3 h. For all the samples with 0 ≤ x ≤ 0.05, the XRD patterns shown are single phase with spinel structure. When 0.07 ≤ x ≤ 0.20, XRD results confirm that the main phase is the cubic spinel phase, and there is a small amount of LaFeO_3_ in the impurity phase. The XRD intensity of LaFeO_3_ increases by increasing the La content, which is due to the La^3+^ ions having bigger ionic radii and very low solubility in spinel lattice [41]. Table 3 indicate XRD data for Co_0.7_Zn_0.3_La_x_Fe_2−x_O_4_ calcined at 800 °C.

**Figure 4:**
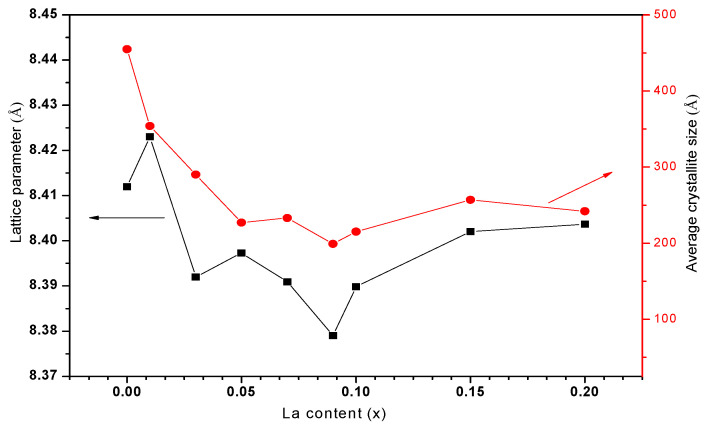
The variation of lattice parameter and average crystallite size for Co_0.7_Zn_0.3_La_x_Fe_2−x_O_4_.

Figure 4 indicate that the lattice parameter tends to decrease with the substitution of La^3+^ ions. It may be that there are secondary phases forming in the grain boundary. Other articles have reported similar results for rare-earth substituted ferrites [10].Average crystallite size tends to decrease with the substitution of La^3+^ ions; similar results are introduced in the other literature [15]. Due to the lower bond energy of Fe^3+^-O^2^ compared to La^3+^-O^2−^, the entry of La^3+^ ions into the lattice to form La^3+^-O^2^ bonds requires more energy. Therefore, for ferrite replaced by La^3+^ ions, more energy is required to complete crystallization and grain growth. Table 3 shows the trend that density decreases with La^3+^ concentration for all samples.The relative atomic weight of La is greater than that of Fe, so the relative molecular weight of the sample increases with the increase in La doping amount. The increase in X-ray density is attributed to an increase in relative molecular weight and a decrease in lattice parameters [42]. The increase in X-ray density also indicates improved densification [43].

**Figure 5:**
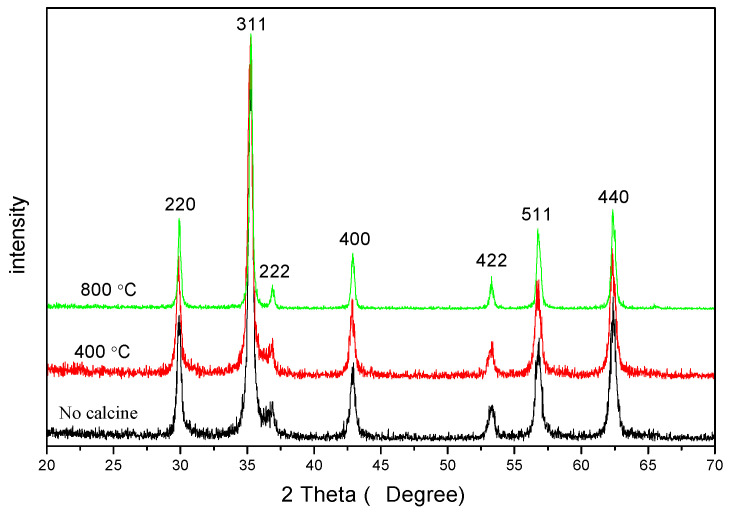
X-ray diffraction patterns of Co_0.7_Zn_0.3_La_0.01_Fe_1.99_O_4_ calcined at different temperatures.

The X-ray patterns of Co_0.7_Zn_0.3_La_0.01_Fe_1.99_O_4_ calcined at different temperatures are displayed in Figure 5 and Table 4. The samples are all single-phase structures of spinel ferrite, and no other impurities were found. The lattice parameters of all samples showed no significant changes, but the average crystallite size showed an increasing trend with the increase in calcination temperature. The diffraction peaks of Co_0.7_Zn_0.3_La_0.01_Fe_1.99_O_4_ without burning are very sharp. The samples without calcination still display excellent crystallinity.

### 2.2. Structures and Grain Sizes

**Figure 6:**
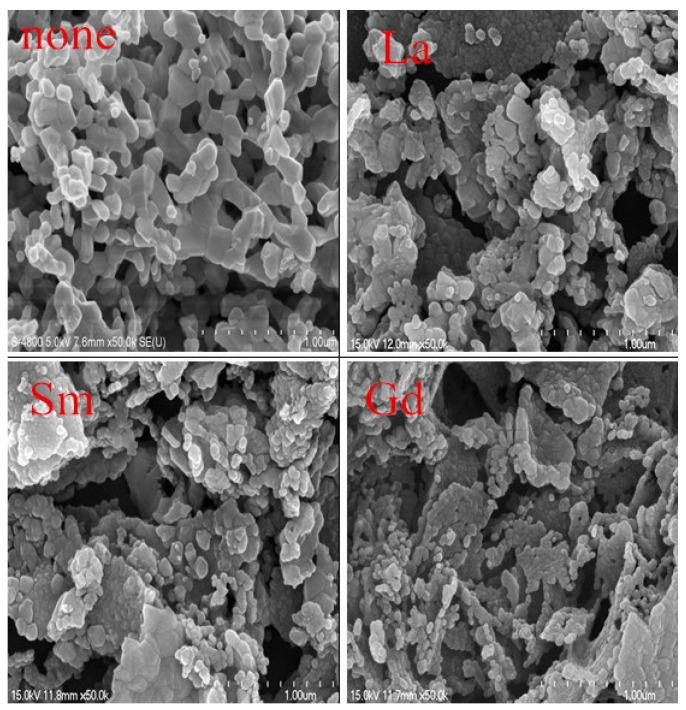
SEM micrographs of CoRE_x_Fe_2−x_O_4_ (x = 0, 0.02; Re = La, Sm, Gd) sintered at 800 °C.

**Figure 7:**
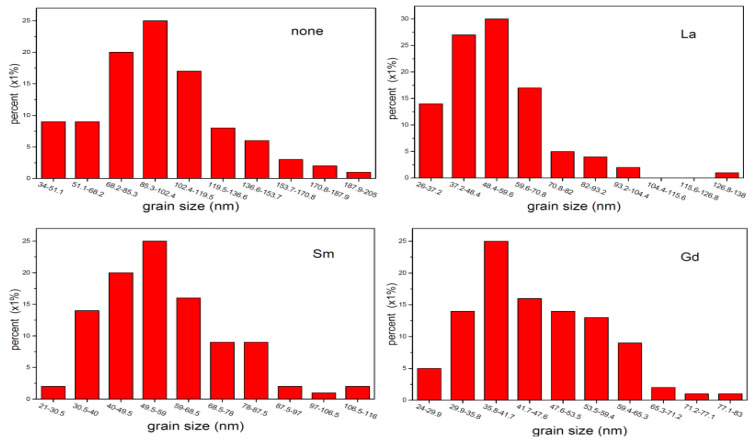
Grain size of CoRE_x_Fe_2−x_O_4_ (x = 0, 0.02; RE = La, Sm, Gd) sintered at 800 °C.

Figure 6 depicts the SEM images of Functional Nanomaterials CoRE_x_Fe_2−x_O_4_ (x = 0, 0.02; RE = La, Sm, Gd) sintered at 800 °C. The sample is well crystallized and displays almost uniform grain sizes. With the substitution of RE^3+^ ions, some cobalt ferrite particles become agglomerated, which may be due to the magnetic interactions between CoRE_x_Fe_2−x_O_4_ particles [11]. Figure 7 shows a histogram of the grain size distribution of CoRE_x_Fe_2−x_O_4_ (x = 0, 0.02; RE = La, Sm, Gd). Statistical methods were used to analyze the average grain size of CoFe_2_O_4_, CoLa_0.02_Fe_1.98_O_4_, CoSm_0.02_Fe_1.98_O_4_, and CoGd_0.02_Fe_1.98_O_4_, which were estimated as approximately 96.26, 54.18, 57.36, and 46.15 nm, respectively. From this, it can be seen that the ferrite samples we prepared are nanoparticles, as their average grain size is less than 100 nm. They are slightly larger than the average crystallite size of X-ray, so each particle is composed of several crystallites [22].

**Figure 8:**
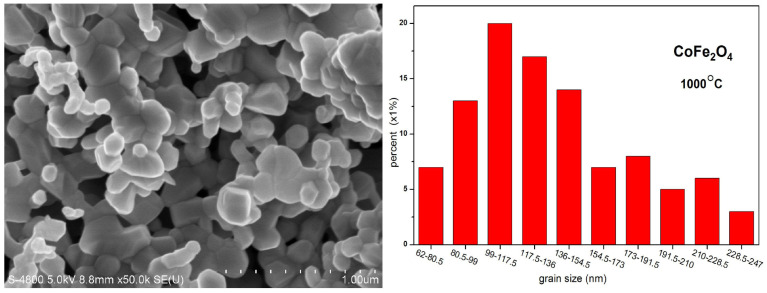
Scanning electron microscopy micrographs and grain size distribution diagram for CoFe_2_O_4_ sintered at 1000 °C.

The SEM micrographs and grain size distribution diagram of CoFe_2_O_4_ sintered at 1000 °C are depicted in Figure 8. The average grain size of CoFe_2_O_4_ ferrite sintered at 1000 °C is estimated to be approximately 137.5 nm, which is larger than the average grain size (96.26 nm) of the cobalt ferrite annealed at 800 °C. This indicates that the average grain size of the CoFe_2_O_4_ sample increases with an increase in the calcining temperature.

**Figure 9:**
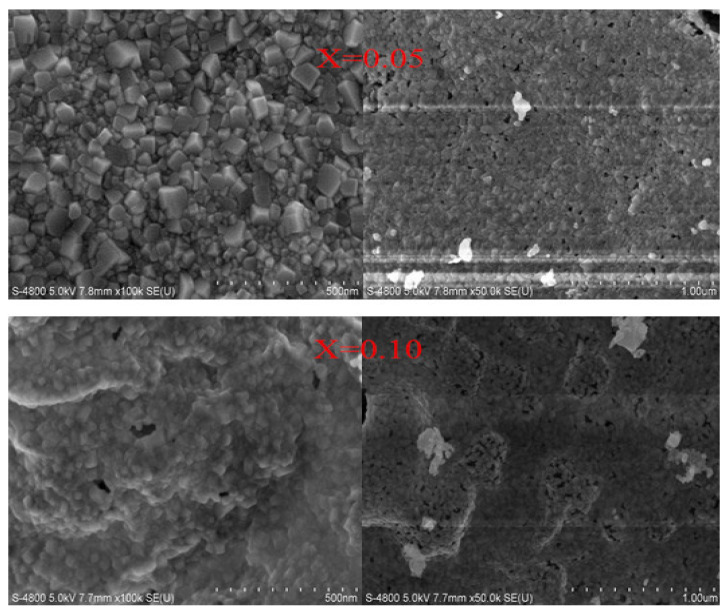
SEM micrographs of Co_0.7_Zn_0.3_La_0.05_Fe_1.95_O_4_ (x = 0.05) and Co_0.7_Zn_0.3_La_0.10_Fe_1.90_O_4_ (x = 0.10) calcined at 800 °C.

**Figure 10:**
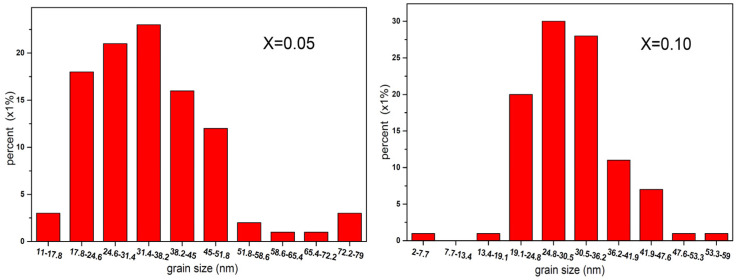
Histogram of grain size distribution of Co_0.7_Zn_0.3_La_0.05_Fe_1.95_O_4_ (x = 0.05) and Co_0.7_Zn_0.3_La_0.10_Fe_1.90_O_4_ (x = 0.10) calcined at 800 °C.

Figure 9 shows the SEM microphotographs of Co_0.7_Zn_0.3_La_x_Fe_2−x_O_4_ (x = 0.05, 0.10) annealed at 800 °C. The sample Co_0.7_Zn_0.3_La_0.05_Fe_1.95_O_4_ we prepared has a relatively uniform grain distribution and good crystallinity, while Co_0.7_Zn_0.3_La_0.10_Fe_1.90_O_4_ has lower crystallinity. Figure 10 shows the histogram of the grain size distribution of Co_0.7_Zn_0.3_La_x_Fe_2−x_O_4_ (x = 0.05, 0.10) ferrites. The significant decrease in the average grain size with increasing La content may be due to the growth of grain being inhibited with LaFeO_3_ [18]. The average grain size of Co_0.7_Zn_0.3_La_0.05_Fe_1.95_O_4_ and Co_0.7_Zn_0.3_La_0.10_Fe_1.90_O_4_ estimated by a statistical method is approximately 35.21 and 30.90 nm, respectively. The average grain size is slightly larger than the average crystallite size of X-ray, so each particle is composed of several crystallites [22].

### 2.3. Room Temperature Mössbauer Spectra

**Figure 11:**
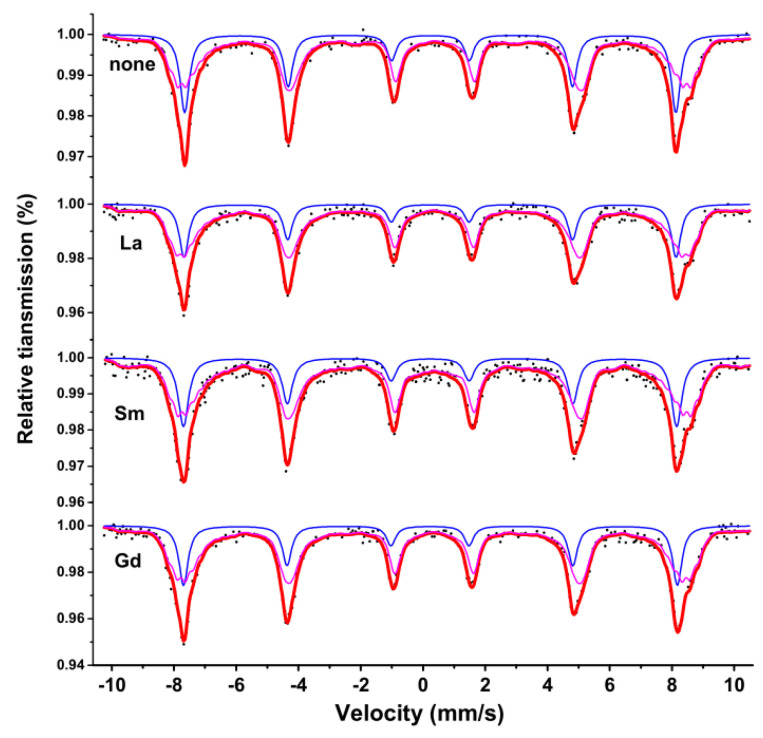
Room temperature Mössbauer spectroscopy curve for CoRE_x_Fe_2−x_O_4_ (x = 0, 0.02; RE = La, Sm, Gd) sintered at 800 °C. (The blue and purple line spectra represent the Mössbauer spectra of iron ions in the A and B lattice, while the red line spectra represent the total Mössbauer spectra).

**Figure 12:**
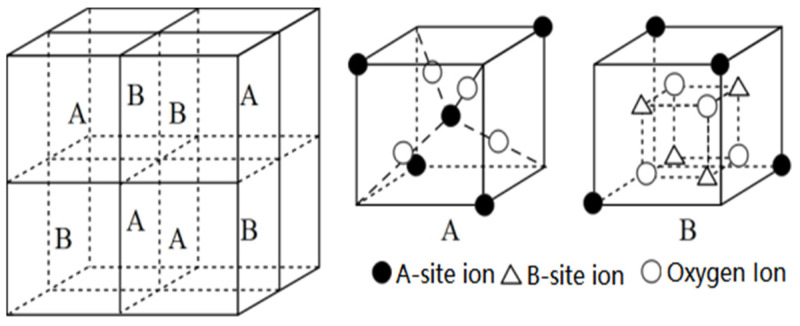
The spatial structure of spinel ferrite cells.

Figure 11 shows the room temperature Mössbauer spectroscopy curve for CoRE_x_Fe_2−x_O_4_ (x = 0, 0.02; RE = La, Sm, Gd) sintered at 800 °C. We used the Mösswinn 3.0 program to fit the measured Mössbauer spectrum, which consists of two sets of six-line peaks.The six linear peaks with a large isomer shift (I.S.) correspond to the iron ions at position B, while the other set of six linear peaks with a smaller isomer shift (I.S.) correspond to the iron ions at position A. This difference is due to the varying internuclear separations between Fe^3+^ and O^2−^ ions [23,24]. The changes in I.S. values due to RE^3+^ substitution are relatively small, and the effect of RE^3+^ doping on the charge distribution around Fe^3+^ is not significant [24]. According to other reports, the I.S. values for Fe^3+^ ions range from 0.1 mm/s to 0.5 mm/s [25]. Therefore, the I.S. values indicate that the iron in our sample is Fe^3+^ ions (Table 5). The magnetic hyperfine field (H) does not appear to be affected by RE^3+^ substitution. Figure 12 displays the spatial structure of spinel ferrite cells. It is possible that micro-substitution is insufficient to significantly alter the H. The quadrupole displacement value in the sample CoRE_x_Fe_2−x_O_4_ is the smallest, indicating good symmetry of the electric field around the atomic nucleus in cobalt ferrite.

**Figure 13:**
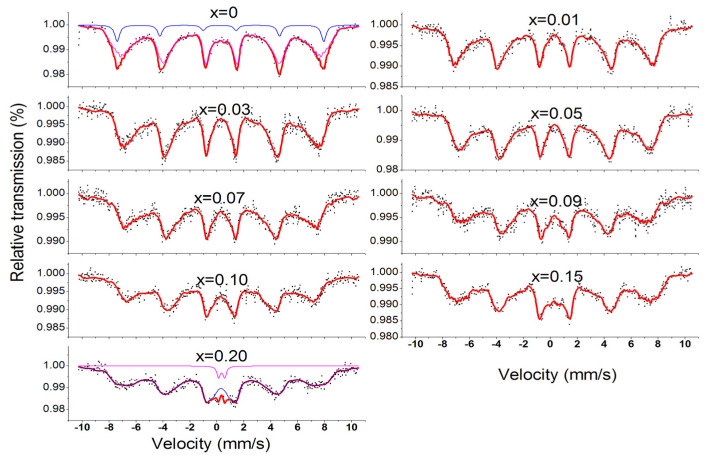
Room temperature Mössbauer spectroscopy curve of Co_0.7_Zn_0.3_La_x_Fe_2–x_O_4_ calcined at 800 °C. (The blue and purple line spectra represent the Mössbauer spectra of iron ions in the A and B lattice, while the red line spectra represent the total Mössbauer spectra).

**Figure 14:**
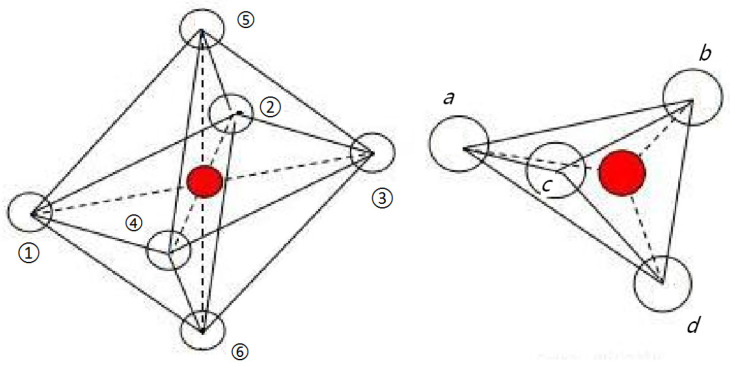
The A site of the tetrahedral lattice and the B site of the octahedral lattice. (1–6 and a–d represent the Oxygen ion).

Figure 13 displays the room temperature Mössbauer spectra of Co_0.7_Zn_0.3_La_x_Fe_2−x_O_4_. The spectra of Co_0.7_Zn_0.3_Fe_2_O_4_, fitted with two sextets of Zeeman-split, are attributed to Fe^3+^ at tetrahedral and octahedral sites, indicating the ferrimagnetic behavior of the samples. The six linear peaks with a large I.S. correspond to the iron ions at position B, while the other set of six linear peaks with a smaller I.S. correspond to the iron ions at position A [20]. When 0.01 ≤ x ≤ 0.15, the Mössbauer spectrum of Co_0.7_Zn_0.3_La_x_Fe_2−x_O_4_ only shows a set of magnetic six-line peaks, corresponding to the six-line peaks at the B site, indicating that Fe^3+^ ions only occupy the octahedral B site [44]. The Mössbauer spectrum of x = 0.20 was fitted with one single sextet and a central paramagnetic doublet, and it shows the relaxation effects features. The A–B exchange interaction decreases with the substitution of nonmagnetic La^3+^ ions, resulting in a decrease in the magnetic hyperfine field value [8]. The large isomer shift (I.S.) value of Fe^2+^ ions is 0.1–0.5 mm/s, while for Fe^3+^, it is 0.6–1.7 mm/s [44]. From the isomer shift (I.S.) values in Table 6, we know that the iron ions in our sample are in the Fe^3+^ state. Figure 14 displays the A site of the tetrahedral lattice and the B site of the octahedral lattice. The quadrupole shift of the magnetic sextet for A and B sites is very small, which indicates that the charge distribution of atomic nuclei is close to spherical symmetry. Mössbauer spectrum of Co_0.7_Zn_0.3_La_0.2_Fe_1.8_O_4_ was fitted with one single sextet and a central paramagnetic doublet, which shows the relaxation effects features [45].

**Figure 15:**
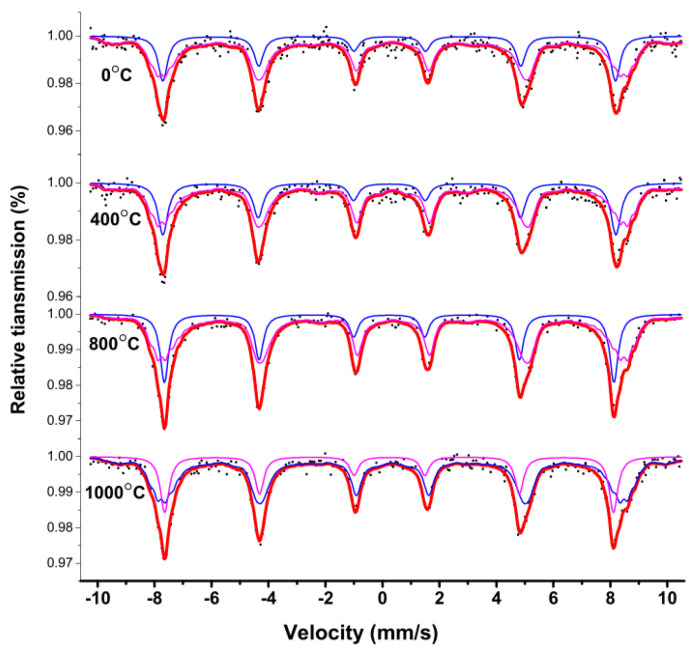
Room temperature Mössbauer spectroscopy curve for CoFe_2_O_4_ unsintered and sintered at 400, 800, and 1000 °C. (The blue and purple line spectra represent the Mössbauer spectra of iron ions in the A and B lattice, while the red line spectra represent the total Mössbauer spectra).

Figure 15 depicts the room temperature (RT) Mössbauer spectroscopy curve for CoFe_2_O_4_ powders that were calcined at 400, 800, and 1000 °C. The spectra comprise two sets of Zeeman sextet splits. Table 7 indicates that there were no significant changes in Mössbauer data for CoFe_2_O_4_ samples that were calcined at various temperatures. The H remained constant with increasing annealing temperature. This may be due to the high crystallinity of the samples without calcination, as observed in the XRD patterns. However, there were changes in the Mössbauer absorption area, indicating that the calcination temperature affected iron ions at the tetrahedral A and octahedral B sites.

**Figure 16:**
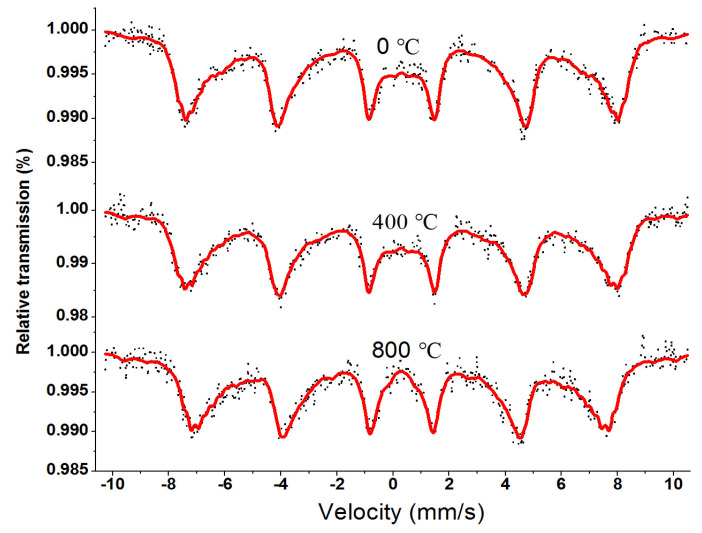
Room temperature Mössbauer spectroscopy curve for Co_0.7_Zn_0.3_La_0.01_Fe_1.99_O_4_ sintered at 0, 400, and 800 °C. (The red line spectra represent theMössbauer spectra of iron ions in the B lattice and the total Mössbauer spectra).

Figure 16 shows the room temperature Mössbauer spectrum of Co_0.7_Zn_0.3_La_0.01_Fe_1.99_O_4_ ferrite. The spectra of all ferrites sintered at different temperatures were fitted using a six-baryon pattern.Table 8 shows no significant change in the Mössbauer parameters of Co_0.7_Zn_0.3_La_0.01_Fe_1.99_O_4_ calcined at different temperatures (unsintered and sintered at 400, 800 °C). The magnetic hyperfine field does not vary with annealing temperature. From the XRD spectrum, this may be due to the good crystallinity of the uncalcined sample.

### 2.4. Magnetic Property of Particles

**Figure 17:**
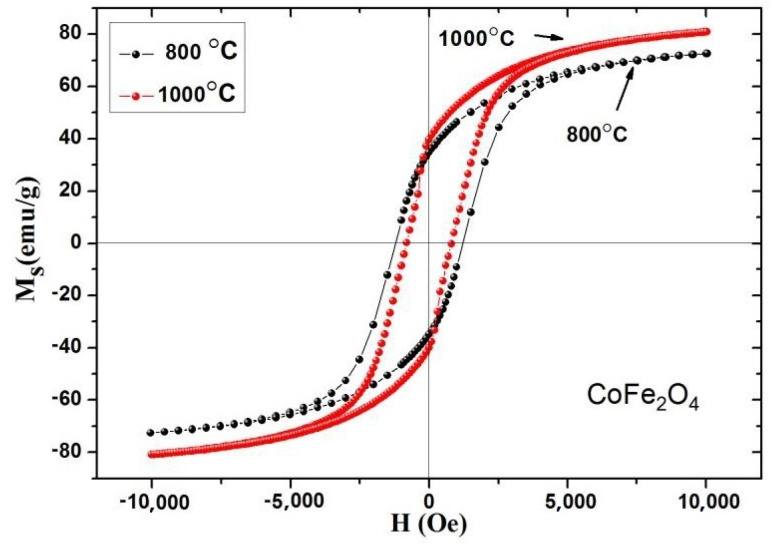
Room temperature magnetic hysteresis curve of CoFe_2_O_4_ sintered at 800 and 1000 °C.

The RT magnetic hysteresis curves of CoFe_2_O_4_ calcined at 800 and 1000 °C are presented in Figure 17. Table 9 shows that the saturation magnetization of CoFe_2_O_4_ increases with the annealing temperature, which is a result of the effect of calcination temperature on particle size [4]. The SEM results reveal that the higher calcination temperature led to greater crystallinity and larger average grain size of the CoFe_2_O_4_ sample. The coercivity of CoFe_2_O_4_ decreases as the annealing temperature increases [10]. The coercivity (*H_C_*) of the sample is related to the grain size (*D*) of the sample. When the sample is a single domain, their relationship is *H_C_ = g* – *h/D^2^*, and when the sample is multi-domain, their relationship is *H_C_ = a* + *b/D*. In the single-domain region, the coercive force increases with the increase in grain size, while in the multi-domain region, the coercive force decreases with the increase in particle diameter. The critical size of cobalt ferrite is approximately 70 nm [13,21]. Based on SEM analysis, the particle size of our sample belongs to a multi-domain region, and the coercive force decreases with the increase in annealing temperature.

**Figure 18:**
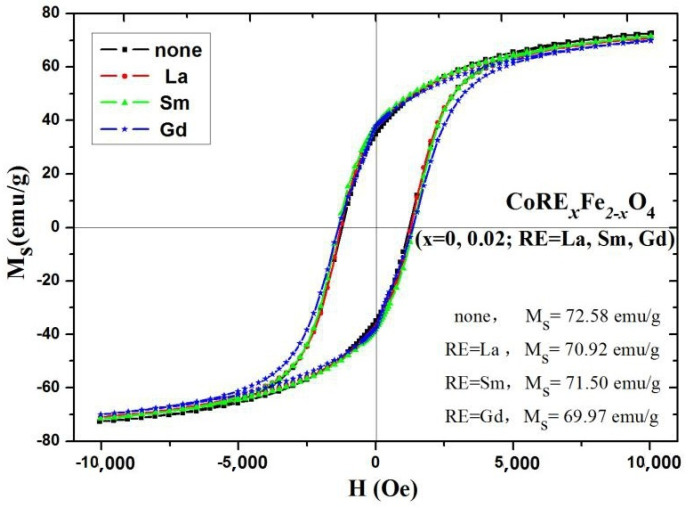
Room temperature magnetic hysteresis curve of CoRE_x_Fe_2−x_O_4_ (x = 0, 0.02; RE = La, Sm, Gd) sintered at 800 °C.

Figure 18 displays the RT magnetic hysteresis curve of CoRE_x_Fe_2−x_O_4_ (x = 0, 0.02; RE = La, Sm, Gd) measured at 295K. The magnetization of CoRE_x_Fe_2−x_O_4_ approaches saturation at 10,000 Oe. The magnetism of rare-earth elements mainly comes from the magnetic moment of their 4f electrons. Due to the inner arrangement of 4f electrons, their magnetic moment is very stable, allowing the magnetism of rare-earth elements to remain stable over a wide temperature range [16,29]. The Gd element has a Curie temperature of 293.2 K, close to RT (295 K) [18]. The magnetic dipoles are arranged disorderly at the temperature of 295 K; therefore, RE ion doping does not contribute to magnetization. 

From Table 10, the substitution of RE^3+^ ions results in a reduction in the saturation magnetization, which can be calculated using the following equation [26,27]:

(2) $$\sigma_{s}=\frac{5585\times n_{B}}{M}$$

where *M* is the molecular mass, and *n_B_* is the Bohr magneton.As RE^3+^ was replaced, the relative molecular mass of CoRE_x_Fe_2−x_O_4_ increased. La^3+^, Sm^3+^, Gd^3+^, Co^2+^, and Fe^3+^ ions have magnetic moments of 0μ_B_, 1.7μ_B_, 7.94μ_B_, 3μ_B_, and 5μ_B_ [11,15,17,18,28], respectively. 

As a result, RE ions (La^3+^, Sm^3+^ and Gd^3+^) are considered nonmagnetic at 295 K. Co^2+^ ions prefer to occupy the B sites, and RE^3+^ ions only occupy the B sites due to their large ionic radius [6,7,18,30,31]. According to the Neel theory, the magnetic moment *n_B_* of (Fe)_A_[CoRE_x_Fe_1−x_]_B_O_4_ can be expressed as [20,29]:

*n_B_* = *M_B_* − *M_A_* = 3 + 5 (1 − *x*) − 5 = 3 − 5*x* (3)

where *M_A_* and *M_B_* are the magnetic moments of the A site and B site, respectively. The substitution of RE^3+^ ions leads to a decrease in the magnetic moment. The theoretical saturation magnetization also decreases, as per Equation (2), which is consistent with experimental results. Table 10 indicates an increase in the coercivity of CoRE_x_Fe_2−x_O_4_ with the addition of RE ions. For CoFe_2_O_4_ ferrite, the large coercivity value is primarily at the B site due to the anisotropy of the cobalt ions [6]. Coercivity is influenced by microstrain, magnetic particle morphology, and magnetic domain size [16,32,33]. Rare-earth ions (RE = La, Sm, and Gd) exhibit stronger magnetocrystalline anisotropy; therefore, the coercivity of cobalt ferrite increases with the substitution of RE^3+^ ions [7,8,21,29]. Additionally, the crystallite size of RE-substituted ferrite decreases with RE^3+^ ion substitution [13,16,34,35]. In our research, the grain size of CoFe_2_O_4_ calcined at 800 and 1000 °C falls within the multi-domain region; the grain size of the sample increases with increasing calcination temperature, so the coercivity decreases as the annealing temperature increases.

**Figure 19:**
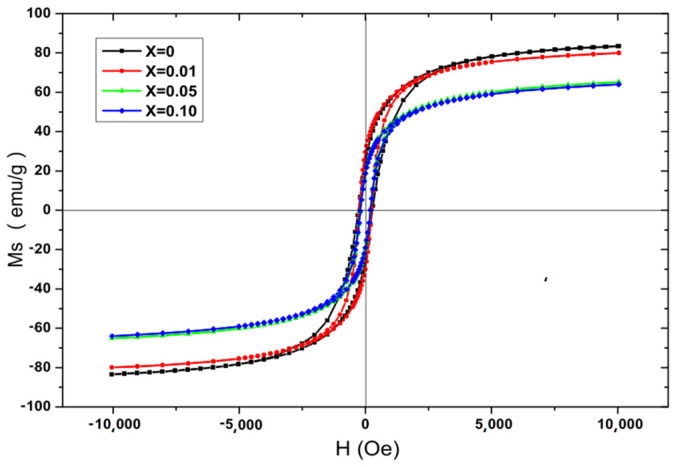
Hysteresis loops of Co_0.7_Zn_0.3_La_x_Fe_2−x_O_4_ calcined at 800 °C.

Figure 19 shows the room temperature hysteresis loop of Co_0.7_Zn_0.3_La_x_Fe_2−x_O_4_. For all samples, the magnetization reached saturation in an external field of 10,000 Oe. 

In Table 11, the saturation magnetization decreases with an increase in La content. The doping of La element affects the magnetic properties of cobalt ferrite.Co^2+^ prefers octahedral sites, and the Zn^2+^ ion prefers tetrahedral sites, which is due to Co–Zn ferrite being an inverted spinel and La^3+^ ions having strong B site preference [14,16], so the cation distribution is (Zn_0.3_Fe)_A_ [Co_0.7_La_x_Fe_1−x_]_B_O_4_ [43,44]. 

**Figure 20:**
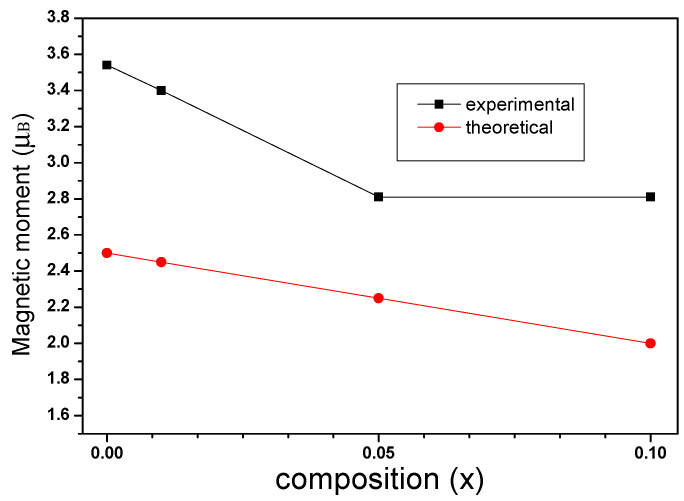
Variation of theoretical and experimental magnetic moment with lanthanum substitution.

According to the two sublattice model of Néel’s theory, the magnetic moment *n_B_* is expressed by [46]:

*n_B_* = *M_B_* − *M_A_* = 3 × 0.7 + 5(1 − *x*) − 5 = 2.5 − 5*x* (4)

where *M_B_* and *M_A_* are the sublattice magnetic moments of the B and A positions. Figure 20 shows the changes in theoretical and experimental magnetic moments as La concentration increases. According to Figure 20, the experimental and theoretical magnetic moments decrease with an increase in La content x. According to Equation (3), the theoretical saturation magnetization decreases with an increase in La content x.

For all samples, the variation in experimental saturation magnetization is well consistent with the theoretical saturation magnetization. As shown in Table 11, the coercivity of Co_0.7_Zn_0.3_La_x_Fe_2−x_O_4_ decreases with increasing La content x. All samples display low coercivity, which shows that they are typical soft ferrites [46,47]. The large coercivity essentially originates from the magnetocrystalline anisotropy of the Co^2+^ ions in the octahedral site [47,48]. The coercivity decreases with increasing lanthanum content, which can be due to the weakening of anisotropy when the Co^2+^ ions migrate to the tetrahedral site. While x = 0.05 and x = 0.10, the coercivity has no significant changes, which may be attributed to the appearance of impurity phases LaFeO_3_ [47,49,50].

## 3. Experimental Method

Magnetic Functional Nanomaterials Co_1−y_Zn_y_RE_x_Fe_2−x_O_4_ (RE (rare-earth) = La,Sm,Gd) were synthesized by the sol–gel combustion method. The synthetic raw material of the sample is analytically pure nitrate Fe(NO_3_)_3_·9H_2_O, M(NO_3_)_2_·6H_2_O (M = Co, Zn, La, Sm, Gd), ammonia (NH_3_·H_2_O), and citric acid (C_6_H_8_O_7_·H_2_O). Add deionized water to citric acid and metal nitrate to form the solution and adjust the pH value to around 7 by adding C_6_H_8_O_7_·H_2_O and NH_3_·H_2_O. Place the mixed solution in a constant temperature water bath at 80 °C, stir it electrically until the gel is dried, dry it further in an oven at 120 °C, and ignite it in air with a small amount of alcohol as an oxidant. After self-ignition, the powder material is annealed in a muffle furnace at a specific temperature. Place the mixed solution in a constant temperature water bath at 80 °C, stir it electrically until the gel is dried, dry it further in an oven at 120 °C, and ignite it in air with a small amount of alcohol as an oxidant. After self-ignition, the powder material is annealed at a specific temperature in a muffle furnace. The various analytical techniques (XRD, SEM, Mössbauer, VSM) were used to determine the following features: the impact of different doping amounts of La, Sm, Gd ion, calcination temperature, and calcination time on the structure, chemical bonding, particle shape and size, magnetic performance, and hyperfine interaction of samples.

## 4. Conclusions

We used the sol–gel combustion method to synthesize Co_1−y_Zn_y_RE_x_Fe_2−x_O_4_ (RE =La, Sm, Gd) nanoparticles. CoFe_2_O_4_ samples calcined at different temperatures (unsintered and sintered at 400 °C, 800 °C, 1000 °C) have good crystallinity. Furthermore, the XRD results indicate that CoRE_x_Fe_2−x_O_4_ ferrites are single spinel-structured. SEM images suggest that the samples are well crystallized, with homogeneously distributed grains and composed of nanoparticles. RT Mössbauer spectroscopy of CoRE_x_Fe_2−x_O_4_ (x = 0, 0.02; RE = La, Sm, Gd) demonstrates ferrimagnetic behavior. Mössbauer spectra of CoFe_2_O_4_ reveal that the magnetic properties are influenced by the calcination temperature. The magnetization curve results suggest that RE ion doping impacts the coercivity and saturation magnetization, allowing the regulation of the sample’s magnetism through RE ion doping. The XRD patterns of Co_0.7_Zn_0.3_La_x_Fe_2−x_O_4_ confirm the main phase is a cubic spinel phase structure along with the appearance of impurity phases LaFeO_3_. It indicates that in spinel lattice, lanthanum has very low solubility. The average grain size decreases significantly with increasing La content. As the La content increases, the average grain size significantly decreases. The Mössbauer spectrum indicates that with the doping of nonmagnetic ions, the sample transitions from a ferrous magnetic state to a relaxed state, while the iron ions in the sample are in the Fe^3+^ state. The saturation magnetization and coercivity decrease with the increase in La content x. The change in coercivity is attributed to the decrease in magnetic crystal anisotropy and the presence of impurity phases LaFeO_3_.

## Figures and Tables

**Table 1:** XRD pattern data for CoFe_2_O_4_ unsintered and sintered at 400, 800, and 1000 °C.

Reaction Temperature (Centigrade)	Lattice Parameter (Å)	Average Crystallite Size (Å)	Density (g/cm^3^)
unsintered	8.41280	361	5.2347
400	8.39149	407	5.2747
800	8.35497	556	5.3468
1000	8.38615	520	5.2847

**Table 2:** XRD pattern data for CoRE_x_Fe_2−x_O_4_ (x = 0, 0.02; RE = La, Sm, Gd.) sintered at 800 °C.

Content (x)	Lattice Parameter (Å)	Average Crystallite Size (Å)	Density (g/cm^3^)
none	8.35497	556	5.3468
La	8.37988	391	5.3341
Sm	8.38629	374	5.3279
Gd	8.40457	402	5.2954

**Table 3:** XRD data for Co_0.7_Zn_0.3_La_x_Fe_2−x_O_4_ calcined at 800 °C.

Content(x)	Lattice Parameter (Å)	Average Crystallite Size (Å)	Density (g/cm^3^)
0	8.41196	455	5.2794
0.01	8.42301	354	5.2769
0.03	8.39198	290	5.3732
0.05	8.39729	227	5.4003
0.07	8.39091	233	5.4500
0.09	8.37899	199	5.5108
0.10	8.38984	215	5.5081
0.15	8.40206	257	5.5771
0.20	8.40366	242	5.6669

**Table 4:** XRD pattern data for Co_0.7_Zn_0.3_La_0.01_Fe_1.99_O_4_ calcined at different temperatures.

Reaction Temperature (Centigrade)	Lattice Parameter (Å)	Average Cryst Size (Å)	Density (g/cm^3^)
unsintered	8.42798	236	5.2678
400	8.43571	227	5.2533
800	8.42301	354	5.2769

**Table 5:** The room temperature Mössbauer spectroscopy data of specimens CoRE_x_Fe_2−x_O_4_ (x = 0, 0.02; RE = La, Sm, Gd) sintered at 800 °C.

Content	Component	I.S. (mm/s)	Q.S. (mm/s)	H (T)	Γ (mm/s)	A_0_ (mm/s)
none	Sextet (1)	0.237	−0.004	48.946	0.360	32.4
	Sextet (2)	0.375	−0.024	45.695	0.322	67.6
La	Sextet (1)	0.228	−0.005	49.023	0.363	24.5
	Sextet (2))	0.342	−0.035	46.381	0.356	75.5
Sm	Sextet (1)	0.229	−0.003	49.146	0.400	27.6
	Sextet (2)	0.368	−0.005	44.827	0.328	72.4
Gd	Sextet (1)	0.231	0.015	49.201	0.357	25.5
	Sextet (2)	0.349	−0.020	46.173	0.342	74.5

**Table 6:** The room temperature Mössbauer spectroscopy data of Co_0.7_Zn_0.3_La_x_Fe_2−x_O_4_ calcined at 800 °C.

Content(x)	Component	I.S. (mm/s)	Q.S. (mm/s)	H(T)	Γ (mm/s)	A_0_ (%)
0	Sextet (1)	0.235	0.055	47.508	0.429	11
	Sextet (2)	0.306	−0.050	38.946	0.338	89
0.01	Sextet (2)	0.276	−0.055	39.416	0.343	100
0.03	Sextet (2)	0.306	−0.002	38.396	0.346	100
0.05	Sextet (2)	0.316	0.001	37.322	0.424	100
0.07	Sextet (2)	0.282	−0.069	36.702	0.334	100
0.09	Sextet (2)	0.296	−0.103	35.311	0.320	100
0.10	Sextet (2)	0.266	−0.058	34.700	0.401	100
0.15	Sextet (2)	0.285	−0.058	34.780	0.332	100
0.20	Sextet (2)	0.311	−0.005	34.476	0.469	97.2
	Doublet	0.350	0.445	-	0.296	2.8

**Table 7:** The Mössbauer spectroscopy data of CoFe_2_O_4_ unsintered and calcined at 400 °C, 800 °C, and 1000 °C.

Reaction Temperature (Centigrade)	Component	I.S. (mm/s)	Q.S. (mm/s)	H (T)	Γ (mm/s)	A_0_ (mm/s)
unsintered	Sextet (1)	0.246	−0.022	49.277	0.401	26.5
	Sextet (2)	0.357	−0.003	45.756	0.341	73.5
400	Sextet (1)	0.241	0.002	49.323	0.422	30.4
	Sextet (2)	0.367	−0.017	45.078	0.319	69.6
800	Sextet (1)	0.237	−0.004	48.946	0.360	32.4
	Sextet (2)	0.375	−0.024	45.695	0.322	67.6
1000	Sextet (1)	0.238	−0.011	48.852	0.366	28.4
	Sextet (2)	0.355	0.0004	45.889	0.338	71.6

**Table 8:** The room temperature Mössbauer spectroscopy data of Co_0.7_Zn_0.3_La_0.01_Fe_1.99_O_4_ calcined at 800 °C.

Reaction Temperature (Centigrade)	Component	I.S. (mm/s)	Q.S. (mm/s)	H (T)	Γ (mm/s)	A_0_ (mm/s)
unsintered	Sextet (2)	0.308	0.001	39.425	0.363	100
400	Sextet (2)	0.300	−0.017	39.123	0.351	100
800	Sextet (2)	0.276	−0.055	39.416	0.343	100

**Table 9:** Magnetic data for CoFe_2_O_4_ sintered at 800 °C and 1000 °C.

Reaction Temperature (Centigrade)	*M_s_* (emu/g)	H_c_ (Oe)	*M_r_* (emu/g)	*n_B_*
800	72.58	1005.33	34.71	3.05
1000	80.89	802.77	37.15	3.40

**Table 10:** Magnetic data for CoRE_x_Fe_2−x_O_4_ (x = 0, 0.02; RE = La, Sm, Gd) sintered at 800 °C.

Sample	*M_s_* (emu/g)	H_c_ (Oe)	*M_r_* (emu/g)	*n_B_*
none	72.58	1005.33	34.71	3.05
La	70.92	1254.00	37.40	3.00
Sm	71.50	1367.66	38.16	3.03
Gd	69.97	1351.25	38.68	2.96

**Table 11:** Magnetic data for Co_0.7_Zn_0.3_La_x_Fe_2−x_O_4_ calcined at 800 °C.

Content (x)	*M_s_* (emu/g)	H_c_ (Oe)	*M_r_* (emu/g)	*n_B_*
0	83.51	301.75	25.00	3.54
0.01	80.02	250.97	29.58	3.40
0.05	65.22	200.90	19.63	2.81
0.10	64.02	200.89	19.04	2.81

## Data Availability

Not applicable.

## Sample Availability

Samples of the compounds are available from the authors.
